# Dose-response associations of maternal prenatal noise exposure duration with antepartum depression status

**DOI:** 10.1186/s12884-023-06200-5

**Published:** 2024-01-02

**Authors:** Jiajin Hu, Borui Liu, Hong Cui, Yilin Liu, Ningyu Wan, Lin Li, Lu Zheng, Xiaochuan Wang, Zhe Yang, Yanan Ma, Caixia Liu, Chong Qiao, Deliang Wen

**Affiliations:** 1 Health Sciences Institute, China Medical University, Shenyang, 110122 China; 2 Research Center of China Medical University Birth Cohort, China Medical University, Shenyang, 110122 China; 3 Division of Chronic Disease Research across the Lifecourse, Department of Population Medicine, Harvard Medical School, Boston, MA 02215 USA; 4 Department of Obstetrics and Gynecology, Shengjing Hospital of China Medical University, China Medical University, Shenyang, 110004 China; 5 Department of Developmental Pediatrics, Shengjing Hospital of China Medical University, China Medical University, Shenyang, 110004 China; 6 Department of Epidemiology and Health Statistics, School of Public Health, China Medical University, Shenyang, 110122 China; 7 Liaoning Key Laboratory of Obesity and Glucose/Lipid Associated Metabolic Diseases, Shenyang, 110122 China

**Keywords:** Dose-response association, Duration of noise exposure, Antepartum depression, Trimester-specific association

## Abstract

**Background:**

Antepartum depression has been reported to be associated with the intensity of maternal prenatal noise exposure; however, the association between noise exposure duration and the development of antepartum depression has not been established. This study aimed to determine the total and trimester-specific association of prenatal noise exposure duration with the development of antepartum depression.

**Methods:**

From May 2018 to June 2021, we recruited 2,166 pregnant women from Shengjing Hospital, northeast China. We used a standardized questionnaire to assess women’s prenatal noise exposure and used the Edinburgh Postnatal Depression Scale to assess pregnant women’s antepartum depression during the 1st -, 2nd -, and 3rd - trimesters. We calculated a cumulative noise exposure score ranging from 0 to 3, with a higher score reflecting higher frequency and longer duration of noise exposure during pregnancy.

**Results:**

Women who were exposed to noise for ≥ 15 min per day had an increased risk of antepartum depression compared with women who were not exposed to noise during pregnancy [odds ratio (OR) = 1.83, 95%CI:1.18, 2.83]. Noise exposure in a specific trimester was associated with higher risk of depression in the same trimester and subsequent trimesters. We observed increases in antepartum depression risk with increasing cumulative noise exposure scores (*P* for trend < 0.05 for all). Pregnant women with the highest scores had the highest risk of antepartum depression during the first (OR = 1.30, 95%CI:1.02, 1.65), second (OR = 1.75, 95%CI:1.23, 2.50) trimesters. Women with a cumulative noise exposure score of 2 had the highest risk of antepartum depression during the third trimester (OR = 1.79, 95%CI:1.14, 2.80), as well as during the whole pregnancy (OR = 1.94, 95%CI:1.14, 3.30).

**Conclusions:**

Maternal prenatal noise exposure duration was positively associated with antepartum depression risk in a dose-response manner. It is necessary to develop strategies by which pregnant women can avoid excessive exposure to noise to prevent antepartum depression.

**Supplementary Information:**

The online version contains supplementary material available at 10.1186/s12884-023-06200-5.

## Background

As a common mental disorder during pregnancy, antepartum depression (APD) has been reported to be linked with adverse birth outcomes, such as preterm birth and low birth weight infants [1, 2], as well as with long-term outcomes after birth such as emotional development disorders and obesity among children [3, 4]. The prevalence of APD in low- and middle-income countries ranges from 12 to 42% [5], which has attracted global public health concern. It is important to identify the risk factors for APD and develop strategies to alleviate APD.

Although environmental factors such as noise pollution have been reported to be linked to depression [6], few studies have examined associations of prenatal noise exposure and APD development. In the Spanish Childhood and Environment Study, more than half of the 2,457 pregnant women were reported to have medium or high annoyance levels at 32 weeks of gestation owing to noise exposure [7]. Another study among 2,018 Chinese pregnant women has reported that, during the third trimester of pregnancy, women exposed to higher levels of noise [≥ 65 dB(A)] were more likely to have antepartum anxiety and depression compared with those exposed to lower levels of noise [< 65 dB(A)] [8]. These studies defined noise based on objective measures of sound, ignoring the fact that noise is a subjective evaluation criterion, and refers to any sound that affects people when resting and working [9]. The self-reported perception of noise by pregnant women will more accurately reflect the disturbance caused by unwanted sound to pregnant women, and will thus be of greater public health significance.

In addition, previous studies [10, 11] mainly assessed the relationship between noise intensity and risk of depression. For example, a large case-control study in Germany compared 77,295 depression cases with 578,246 control subjects, and found that for every 10 dB increase in road traffic noise, the risk of depression increased by 4% [10]. Another cross-sectional study in Sweden including 627 households reported that, for every 2.5 dB(A) increase of noise from wind turbines, respondents were 1.87 times more likely to be annoyed [11]. Compared with the noise intensity, the cumulative duration of noise exposure may have a more significant impact on the development of depression in pregnant women, based on the fact that pregnant women are more likely to be exposed to longer-term low-intensity noise rather than high intensity noise [12]. Development of maternal depression is gradually aggravated across the three trimesters, and the morbidity of APD was found to be highest in the third trimester [13]. To examine the dose-response associations of prenatal noise exposure duration with APD development may help to identify target intervention strategies to prevent APD in later trimesters.

To address the knowledge gaps, we used the data from a prospective pre-birth cohort study in China to investigate the association between noise exposure during pregnancy and APD. We hypothesized a dose-response relationship between the cumulative duration of noise during pregnancy and risk of APD.

## Methods

### Study population

**Fig. 1:**
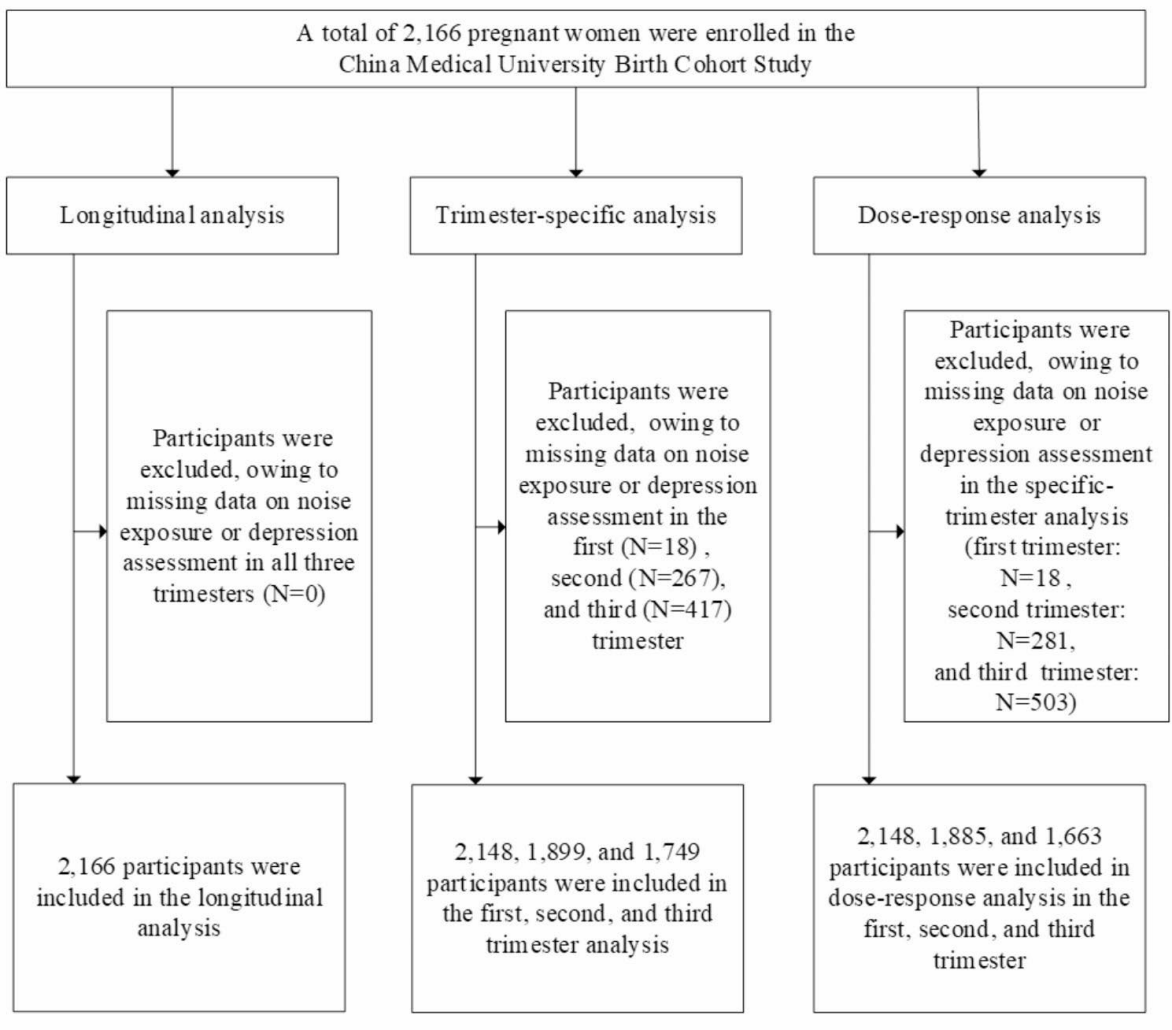
Flow chart

We established a prospective pre-birth cohort study, the China Medical University Birth Cohort Study, in northeast China, to examine the associations between prenatal factors and maternal and child health outcomes. The study design has been published previously [14]. In brief, we recruited pregnant women who were at gestational age < 14 weeks, and had no plan to move out of Shenyang in the next three years. A total of 2,166 pregnant women were enrolled in the study from May 2018 to June 2021. We conducted face-to-face interviews and collected the women’s socio-demographic, environmental, behavior and clinical information at the first (9.4 ± 4.1 gestational weeks), second (23.2 ± 2.8 gestational weeks) and third trimesters (31.5 ± 2.2 gestational weeks). In the analysis, we included in the longitudinal analysis all of the 2,166 participants who had at least one assessment of noise exposure and statement regarding depression. In the trimester-specific analysis, we included 2,148, 1,899 and 1,749 women in the first, second and third trimesters, respectively, who had full assessments of noise exposure and depression in the corresponding trimester. In the dose-response analysis, we included 2,148, 1,885, and 1,663 women who had complete assessments in the current and previous trimesters. (Fig. 1). All participants provided written informed consent.

### Noise exposure

We used a standardized questionnaire to assess the noise exposure of the pregnant women. The questionnaire has been previously used to assess women’s prenatal noise exposure in the Born in Shenyang Cohort Study, a prospective pre-birth cohort study we conducted in the same area [15]. The questionnaire can well assess women’s prenatal noise exposure duration, which has been previously demonstrated as a predictor for women’s postpartum depression development according to our unpublished results. Noise has been defined as unwanted sounds [9, 16]. Trained research stuff asked pregnant women to report their daily noise exposure at 1st -, 2nd -, and 3rd -trimester visits, with the question “how long have you been exposed to noise which bothered you when resting, working, or studying during the last trimester?”. The question had six feedback frequency categories: never; less than 15 min per day; 15 min to less than 1 h per day; 1 h to less than 2 h per day; 2 h to less than 3 h per day; at least 3 h per day. We further combined the six categories into a three-category variable (never; less than 15 min per day; at least 15 min per day) and into a binary variable (never vs. ever) to improve statistical power in different statistical models. We also computed noise exposure scores by calculating the cumulative noise exposure experience (in dichotomous form) across the three trimesters and limited the score to range from 0 to 3. A higher score reflected higher frequency and longer duration of noise exposure during pregnancy.

### Measurement of antepartum depression

We used the Edinburgh Postnatal Depression Scale (EPDS) to assess pregnant women’s APD during the 1st -, 2nd -, and 3rd -trimester visits. The EPDS has been shown to have good reliability and validity when screening for APD in China [17]. The EPDS consists of 10 items which assessed participants’ depressive symptoms during the last week. Each item was rated by a score of 0–3, with the total score ranging from 0 to 30. The total score of the scale was calculated by summing the scores of all items, with higher scores indicating more severe symptoms. We defined APD as an EPDS score ≥ 10 according to a previous study that recommended the cutoff score of 9.5 when using the EPDS to screen APD among Chinese pregnant women [17]. In the sensitivity analysis, we also used the cutoff score of 9 to check the robustness of the results. In the repeated measures analysis, we calculated women’s average EPDS score across three trimesters, and defined women’s APD status across the pregnancy according the average score. In the trimester-specific analysis and the dose-response analysis, APD status in different trimester was evaluated according to the EPDS score during the corresponding trimester.

### Covariates

We collected the maternal social-economic and behavioral information using standard questionnaires, including the women’s age (in years), ethnicity (Han vs. others), education attainment (high school or below vs. college or above), annual household income (< ¥ 50,000 and ≥ ¥ 50,000); parity (primipara vs. multipara); marital status (married vs. single); and smoking status during pregnancy (yes vs. no). We used the Pittsburgh Sleep Quality Index (PSQI) to assess the women’s sleep quality during each of the three trimesters. The PSQI is a 19-item self-report questionnaire designed to assess sleep disorders in the clinical population over the past month and the PSQI has shown good reliability and validity in Chinese population [18, 19]. The PSQI index ranges from 0 to 21 and a higher index represents poorer sleep quality [20, 21]. We used a Chinese version of the Pregnancy Stress Scale (PSS) to assess the level of psychological stress in the pregnant women during each trimester. The scale has been widely used in relevant research on pregnant women, and has shown good reliability and validity in Chinese population [22]. The scale includes 30 items on a 4-point scale (total scores ranging from 0 to 90), with higher scores indicating higher stress levels. The pre-pregnancy body mass index (BMI) was based on maternal self-report of pre-pregnancy weight and measured by study personnel during the enrollment visit. Height was measured to the nearest 0.1 cm with a ruler and weight to the nearest 0.01 kg using calibrated electronic scales. The pre-pregnancy BMI of women was calculated using self-reported pre-pregnancy weight (in kg) and measured height (in meters) and categorized into underweight (BMI < 18.5 kg/m^2^), normal weight (18.5 kg/m^2^ ≤ BMI < 24 kg/m^2^) and overweight/ obesity (BMI ≥ 24 kg/m^2^).

### Statistical analysis

We used the *t*-test and ANOVA to compare the sociodemographic characteristics between pregnant women with and without APD. The Pearson’s correlation coefficient was used to assess the correlation between depression scores during each trimester. We used three different statistical models to examine the association between maternal noise exposure and APD. First, we used a generalized estimation equation model to examine the longitudinal association between maternal APD and noise exposure across first, second and third trimesters. Second, we used a linear regression model to examine the independent association between noise exposure in each trimester and APD scores in the same and subsequent trimesters. Third, we used multivariable logistic regression models to estimate the odds ratio (OR) of noise exposure score to APD in different trimesters.

We conducted crude and adjusted models, as follows: model 1: crude model; model 2: adjusted for age at enrollment, annual household income, ethnicity, marital status, education attainment, pre-pregnancy BMI and parity; Model 3: model 2 + smoking status and sleep quality score; Model 4: model 3 + maternal stress. We considered these confounding factors and incorporate confounding factors into co-variables because they were either demographic characteristics or behavioral factors previously reported to be independently associated with APD risk [23]. Depression in the first trimester is important for the development of the whole pregnancy [24], and we excluded pregnant women who were already depressive at the time of enrollment to examine the independent association of the influence of noise exposure in a sensitivity analysis. To examine the independent association between trimester-specific noise exposure and APD scores in subsequent trimesters (for example, the association between noise exposure in the first trimester and depression scores in the second and third trimesters), we also adjusted the gestational depression score at the same time as the noise exposure assessment. In multiple logistic regression models, we investigated the association of APD risk in the 1st, 2nd and 3rd trimesters and the whole pregnancy with the cumulative noise exposure scores.

All statistical analyses were performed using Stata S.E. version 16 (Stata Corp, Texas, United States).

## Results

### Participants’ characteristics

The prevalence of APD was 21.9%, 16.2% and 16.8% during the 1st, 2nd and 3rd trimester, respectively. The Pearson’s correlation coefficient of depression scores across the three trimesters ranged from 0.52 to 0.73 (*P* < 0.01 for all) (Table S1). Compared with women who had an average APD score < 10, women who had an average APD score ≥ 10 were more likely to have a higher PSS score and sleep quality index. There was no significant difference in age, ethnicity, level of education, household income per year, parity, marital status, pre-pregnancy BMI or smoking status between women with APD scores ≥ 10 and < 10 (Table 1).

**Table 1:** Characteristics of participants according to antepartum depression status in the CMUBC study

Characteristic	Average antepartum depression score across three trimesters (n = 2,166)		
	EPDS < 10	EPDS ≥ 10	*P* value
Age at enrollments (years)	31.5 ± 4.1	31.3 ± 4.2	0.31
Ethnicity			0.91
Han	1,523 (92.5)	255 (92.7)	
Others	123 (7.5)	20 (7.3)	
Educational attainment			0.50
High school or below	261 (14.2)	41 (12.8)	
College or above	1,572 (85.8)	279 (87.2)	
Household income per year, CNY			0.86
< 50,000	639 (40.0)	110 (40.6)	
≥ 50,000	958 (60.0)	161 (59.4)	
Parity			0.97
Primipara	773 (67.2)	146 (67.3)	
Multipara	378 (32.8)	71 (32.7)	
Marital status			0.16
Married	1,814 (99.2)	313 (98.4)	
Single	14 (0.8)	5 (1.6)	
Stress score			
First trimester	39.6 ± 10.0	51.4 ± 14.1	< 0.001
Second trimester	39.0 ± 9.4	54.2 ± 13.4	< 0.001
Third trimester	38.5 ± 9.3	52.9 ± 13.5	< 0.001
Stress score categories			< 0.001
< 38.5	1,022 (55.4)	37 (11.6)	
≥ 38.5	822 (44.6)	283 (88.4)	
Pre-pregnancy BMI categories, kg/m^2^			0.23
< 18.5	226 (12.4)	50 (15.8)	
18.5- < 24.0	1,042 (57.1)	177 (55.8)	
≥ 24.0	558 (30.6)	90 (28.4)	
Smoking status			0.07
No	1,780 (97.2)	299 (95.2)	
Yes	52 (2.8)	15 (4.8)	
Pittsburgh sleep index			
First trimester	5.1 ± 2.4	6.6 ± 2.8	< 0.001
Second trimester	4.8 ± 2.5	7.0 ± 3.0	< 0.001
Third trimester	5.4 ± 2.9	7.7 ± 3.0	< 0.001

Abbreviations: CMUBC, China Medical University Birth Cohort; EPDS, Edinburgh postnatal depression scale; CNY, Chinese Yuan; BMI, body mass index

### Longitudinal associations between noise exposure during pregnancy and antepartum depression

In the longitudinal analysis, women who were exposed to noise for ≥ 15 min per day had an increased risk of APD compared with women who reported no exposure to noise during pregnancy (OR = 1.83, 95%CI:1.18, 2.83) (Table 2). In sensitivity analyses, the association between noise exposure and APD generally remained stable after further excluding participants who were depressed at their enrollment visit (OR = 2.63, 95%CI:1.54, 4.52) (Table S2), or using the EPDS cutoff value of 9 to define APD (OR = 2.03, 95%CI:1.35, 3.05) (Table S3).

**Table 2:** Repeated measures analysis: association of antepartum depression with noise exposure across pregnancy among 2,166 participants

Antepartum depression	Nosie exposure, OR (95% CI)			*P* for trend
	Never	< 15 min per day	≥ 15 min per day	
Model 1	1.00 (Ref.)	2.00 (1.62, 2.48)	3.49 (2.73, 4.48)	< 0.001
Model 2	1.00 (Ref.)	1.96 (1.44, 2.66)	3.30 (2.31, 4.72)	< 0.001
Model 3	1.00 (Ref.)	1.86 (1.35, 2.57)	3.15 (2.14, 4.63)	< 0.001
Model 4	1.00 (Ref.)	1.27 (0.89, 1.80)	1.83 (1.18, 2.83)	< 0.01

Model 1: crude model

Model 2: adjusted for age at enrollment, household income, ethnicity, marital status, level of education, pre-pregnancy BMI, and parity

Model 3: Model 2 + smoking status and sleep quality score throughout pregnancy

Model 4: Model 3 + stress throughout pregnancy

Abbreviations: OR, odds ratio; CI, confidence interval; BMI, body mass index

### Trimester-specific associations between noise exposure during pregnancy and antepartum depression

**Fig. 2:**
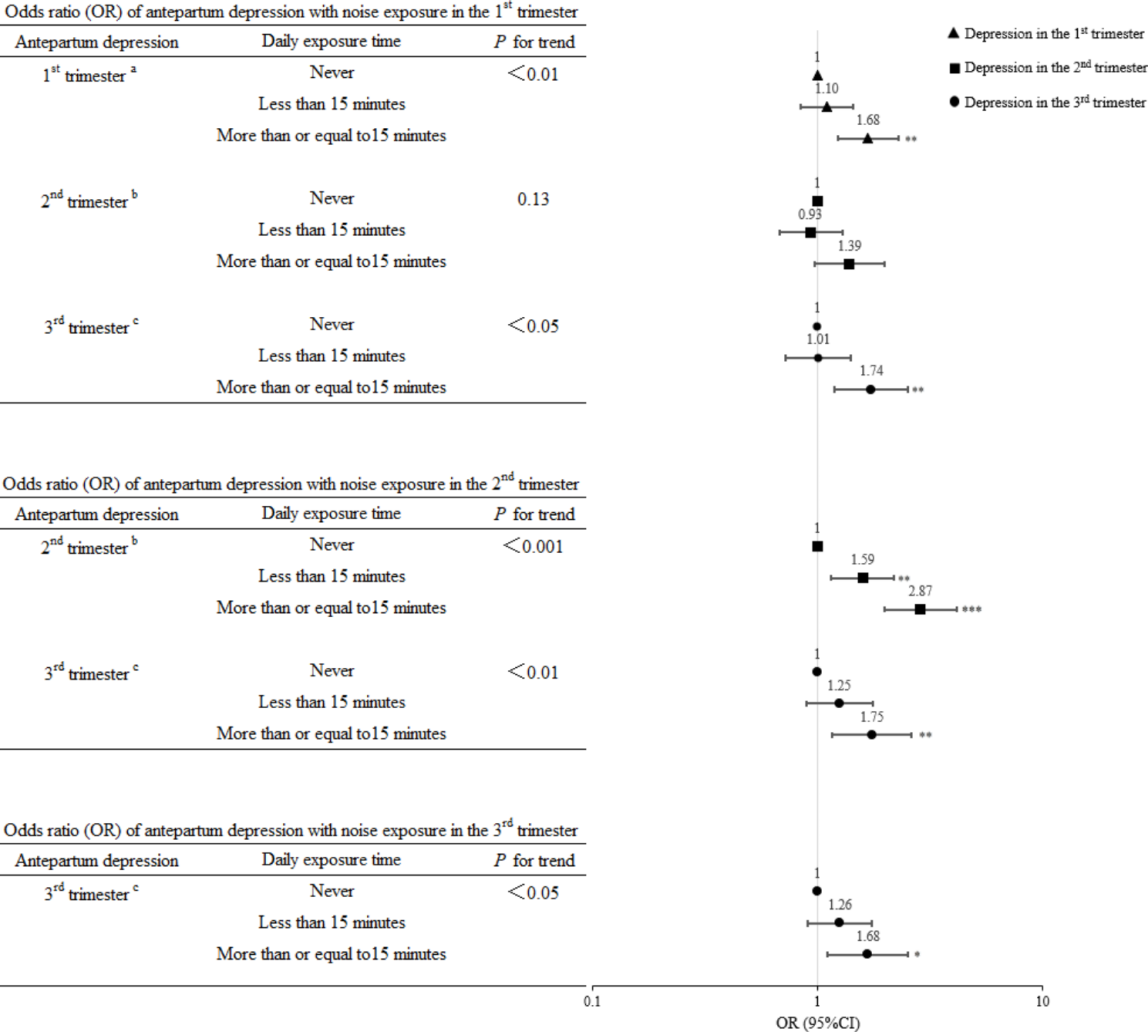
Trimester-specific analysis: associations between antepartum depression status and noise exposure in each trimester ^a^Adjusted for age at enrollment, household income, ethnicity, marital status, level of education, pre-pregnancy BMI, parity, smoking status, sleep quality score, and stress in the 1st trimester. ^b^Adjusted for age at enrollment, household income, ethnicity, marital status, level of education, pre-pregnancy BMI, parity, smoking status, sleep quality score, and stress in the 2nd trimester. ^c^Adjusted for age at enrollment, household income, ethnicity, marital status, level of education, pre-pregnancy BMI, parity, smoking status, sleep quality score, and stress in the 3rd trimester. Abbreviations: OR, odds ratio; CI, confidence interval; BMI, body mass index ^***^P < 0.001; ^**^P < 0.01; ^*^P < 0.05

Figure 2 presents the trimester-specific associations between APD risk and noise exposure. Noise exposure in a specific trimester was associated with higher APD risk in the same trimester and subsequent trimesters. For example, exposure to noise for ≥ 15 min in the 1st trimester was positively associated with APD risk in the 1st (OR = 1.68, 95%CI:1.24, 2.29), 2nd (OR = 1.39, 95%CI:0.97, 1.98) and 3rd trimesters (OR = 1.74, 95%CI:1.19, 2.53) when compared with participants who reported no exposure to noise. In general, further adjustment for depression status in the previous trimesters (Figure S1) or using the EPDS cutoff value of 9 to define APD (Figure  S2) did not change the results.

### Association between cumulative noise exposure scores during pregnancy and antepartum depression

**Fig. 3:**
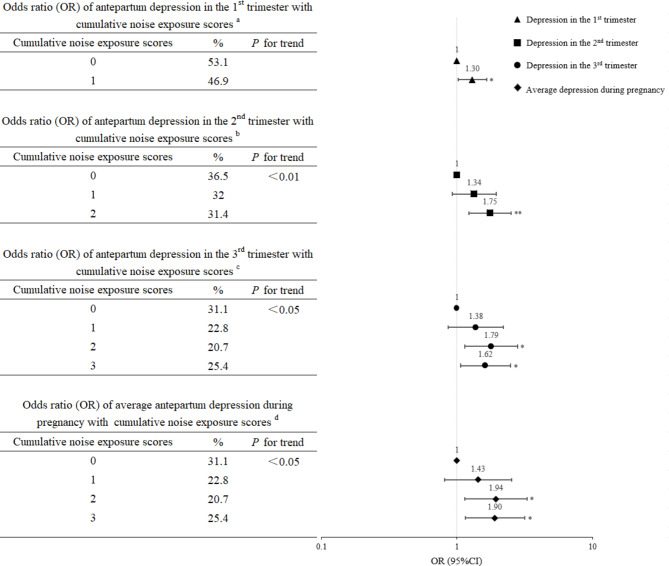
Dose-response analysis: associations between cumulative noise exposure scores and depression status across three trimesters ^a^Adjusted for age at enrollment, household income, ethnicity, marital status, level of education, pre-pregnancy BMI, parity, smoking status, sleep quality score, and stress in the 1st trimester. ^b^Adjusted for age at enrollment, household income, ethnicity, marital status, level of education, pre-pregnancy BMI, parity, smoking status, sleep quality score, and stress in the 2nd trimester. ^c^Adjusted for age at enrollment, household income, ethnicity, marital status, level of education, pre-pregnancy BMI, parity, smoking status, sleep quality score, and stress in the 3rd trimester. ^d^Adjusted for age at enrollment, household income, ethnicity, marital status, level of education, pre-pregnancy BMI, parity, smoking status, average sleep quality score, and average stress throughout pregnancy. Abbreviations: OR, odds ratio; CI, confidence interval; BMI, body mass index ^**^P < 0.01; ^*^P < 0.05

Figure 3 presents the associations between the cumulative noise exposure scores and depression status. We observed increases in depression risk during specific trimesters and the whole pregnancy with increasing cumulative noise exposure scores (*P* for trend < 0.05 for all). Pregnant women with the highest cumulative noise exposure scores had the highest risk of APD during the 1st (OR = 1.30, 95%CI:1.02, 1.65), 2nd (OR = 1.75, 95%CI:1.23, 2.50) trimesters. Women with a cumulative noise exposure score of 2 had the highest risk of APD during the 3rd trimester (OR = 1.79, 95%CI:1.14, 2.80), as well as during the whole pregnancy (OR = 1.94, 95%CI:1.14, 3.30). In sensitivity analysis, pregnant women with the highest cumulative noise exposure scores had the highest risk of APD during the 1st, 2nd and 3rd trimesters, as well as the whole pregnancy (Figure S3).

## Discussion

To our knowledge, this is the first study to examine the dose-response association of prenatal noise exposure duration with antepartum depression status. In this prospective pre-birth cohort, we observed that the duration of maternal daily noise exposure during pregnancy was positively associated with depressive symptoms in specific trimesters and throughout pregnancy. We also observed a dose-response relationship and higher antepartum depression risk during pregnancy with increasing cumulative noise exposure scores across the three trimesters.

In our study population, the prevalence of APD was highest during the first trimester, and decreased at the second and the third trimester, which is inconsistent with previous studies reported that APD increased by trimester [13]. One possible reason could be that our study sample has relatively higher rates of primipara, who are lack of experience with pregnancy and caring infants [25]. That may cause high rate of APD at the beginning of pregnancy and the rate of APD may decreased with the psychological adjustment to pregnancy in later trimesters. Our study indicated that noise exposure during pregnancy was positively associated with the risk of APD, which is consistent with previous studies [8, 26, 27]. A cross-sectional study reported that pregnant women exposure to higher noise intensity [≥ 65 dB(A)] had a 71% increased risk of APD when compared with women with a low level of noise exposure [< 65 dB(A)] [8]. In a Canadian longitudinal study, pregnant women exposed to 70 dB(A) of noise were reported to have an increased risk of depression compared with those exposed to 50 dB(A) of noise [26]. In a European study, a 3 dB(A) reduction in noise intensity reduced the population-attributable fraction for depressive disorders from 3.5 to 2.7% [27]. However, these studies mainly examined the health effect of noise intensity based on objective measured values of environmental sound [8, 26, 27]. Noise has been defined as unwanted sounds of any intensity which bother people when resting and working [9]. Peoples’ sensitivity to noise might be an indicator of vulnerability to environmental stressors, so that highly sensitive people may be more prone to developing depression when exposed to environmental noise [28]. Maternal perception of noise was self-reported, which could better reflect the health association of environmental sound exposures with women’s mental health. In our study, further adjusted for women’s sleep quality and stress attenuated the association between noise exposure and APD risk. This is in line with previous studies that reported women’s sleep quality and stress status may also influence APD [22, 29]. For example, in a Peru cohort, pregnancy women with sleep disorders were 2.74 times more likely to develop APD than their counterparts without sleep disorders [29]. However, the association between noise exposure and APD remains significant in the full adjusted models, demonstrated the independent association of noise exposure and APD development.

Our study reported that adverse noise exposure in a specific trimester was associated with increased APD risk not only in the current trimester, but also in the subsequent trimesters. This demonstrated the long-term association of noise pollution with APD, which is consistent with previous research [26, 30]. In the Gutenberg Health Study, the annoyance level at baseline was significantly correlated with participants’ depression and anxiety symptoms 5 years later [30]. Another study reported that exposure to noise pollution during pregnancy was associated with the risk of later hospitalization for depression, and residential noise during pregnancy affected the long-term mental health of pregnant women [26]. In our study, after adjusting for the depression status in the current trimester, the health association between noise exposure and APD in later trimesters was still positive, which suggests that there is continuity in the impact of noise on depression. These findings highlight the importance of noise prevention in early trimesters to minimize the potential long-term adverse effects on women’s mental health throughout pregnancy.

Our study reported that longer duration and repeated exposure to noise across the three trimesters was correlated with a higher risk of APD in a dose-response manner, which extended previous study evidence on the dose-response association between noise intensity and depression [11, 31]. A cross-sectional study reported that, at sound levels exceeding 35 dB(A) (about the noise of a refrigerator working), the proportion of those annoyed by wind turbine noise outdoors increased with higher sound level [11]. For example, 20% of the 40 respondents living within this exposure at sound category 37.5–40 dB(A) and 36% of the 25 respondents above sound category 40 dB(A) were very annoyed. A Dutch study reported a positive correlation between noise exposure levels and participants’ annoyance score; specifically, every decibel increase in noise was associated with a 3-point increase in annoyance score on a 100-point annoyance scale [31]. Pregnant women may take noise mitigation measures (such as noise protection windows) and behavioral prevention measures (such as closing windows, wearing earplugs) when exposed to high intensity noise [32], but they may not pay enough attention to low intensity continuous noise in the working environment. Our findings highlighted the health influence of the cumulative exposure to noise on women’s mental health.

Previous studies have shown that dysregulation of the hypothalamic-pituitary-adrenal (HPA) axis is significantly associated with multiple mental health disorders such as depression [33]. Noise exposure can cause mental stress. If the exposure persists over a period of time, the cognitive and emotional state of stress may cause a pathophysiological cascade. For example, noise stress-induced autonomic disturbance and sympathoadrenal activation may lead to elevated circulating stress hormone levels and subsequent oxidative stress-induced endothelial dysfunction [34, 35]. Endothelial dysfunction may contribute to depression by inducing HPA axis hyperactivity and activation of the inflammatory response [36, 37]. Therefore, noise exposure may induce the release of stress hormones and disrupt hormonal rhythms by activating the HPA axis [33]. The mechanisms underlying the dose-response relationship between noise and APD remain unclear. Previous study has shown that acute exposure to night-time aircraft noise dose-dependently impairs endothelial function [38]. Oxidative stress may be involved in mediating noise-induced depression [39]. Future studies are needed to explore the mechanism of the dose-response relationship between noise exposure duration and APD.

The main strength of this study was the repeated measurements of noise exposure and depressive symptoms in pregnant women, which enabled us to examine the trimester-specific associations between APD and noise exposure and the dose-response associations between APD risk and the cumulative noise exposure duration across three trimesters. In addition, we were able to adjust for a series of confounders, such as prenatal stress and sleep quality, which is important because these two factors have been reported to be independently associated with APD [40, 41].

Our study also had several limitations. First, our noise exposure data were self-reported, and therefore may not reflect the objective noise intensity. While subjective noise measurements may not be as accurate as objective measurements, subjective noise annoyance (reflecting inter-individual variability in noise perception) might better capture certain health associations of noise exposure, as demonstrated by recent studies [42, 43]. Noise is an unwanted sound so the human experience of noise is not just about its intensity level. The self-reported noise annoyance was an important predictor of depression, and personal attitudes to noise may influence noise annoyance [44]. Second, the EPDS is a screening tool for depression, and cannot be used in diagnosis. However, previous studies have shown that it is a useful tool for evaluating depressive status in pregnant women and has good reliability and validity [17]. Third, there may be confounding factors that were not adjusted for in this study, such as physical activity and air pollutants which are associated with the development of APD [45, 46]. Fourth, we do not have information on the treatment of APD during pregnancy, which may influence the prevalence and development of APD in our study population. However, the safety of antidepressant use for pregnant women and infants remain inconclusive. Some researches recommendations that pregnant women are not recommended to take any antidepressant drug treatment in general, because it may lead to adverse pregnancy outcomes, such as fetal malformations, miscarriage, preterm birth or low birth weight [47, 48]. Other researches have shown that the risk of preterm birth and low birth weight are similar between antidepressants and untreated depression [49, 50]. Thus, the limitation is less likely to affect the present results. Fifth, in the present analysis, we do not have the information of the source of noise (e.g., traffic noise). That limited us on examining the noise types on the development of APD, which we will address in the future studies. Finally, our study was based on a regional population, which may limit the generalizability of our findings.

## Conclusions

In this prospective pre-birth cohort study, we found that noise exposure during pregnancy was positively associated with the risk of APD. Adverse noise exposure in a specific trimester was associated with increased APD risk not only in the current trimester but also in the subsequent trimesters. The results also indicated that longer duration and repeated exposure to noise across the three trimesters was correlated with higher risk of APD in a dose-response manner. Our findings highlighted the detrimental association between cumulative exposure to noise and maternal prenatal mental health. Strategies should be developed to avoid excessive maternal exposure to noise in order to prevent APD.

### Electronic supplementary material

Below is the link to the electronic supplementary material.

Supplementary Material 1

## Data Availability

The datasets used and analyzed during the current study are available from the corresponding author upon request.

## List of abbreviations

APD: Antepartum depression
BMI: Body mass index
CMUBC: China Medical University Birth Cohort Study
EPDS: Edinburgh Postnatal Depression Scale
HPA: Hypothalamic-pituitary-adrenal
OR: Odds ratio
PSQI: Pittsburgh Sleep Quality Index
